# Identification of SNPs and Candidate Genes Associated with Monocyte/Lymphocyte Ratio and Neutrophil/Lymphocyte Ratio in Duroc × Erhualian F_2_ Population

**DOI:** 10.3390/ijms25179745

**Published:** 2024-09-09

**Authors:** Jiakun Qiao, Minghang Xu, Fangjun Xu, Zhaoxuan Che, Pingping Han, Xiangyu Dai, Na Miao, Mengjin Zhu

**Affiliations:** 1 Key Lab of Agricultural Animal Genetics, Breeding, and Reproduction of Ministry of Education, Huazhong Agricultural University, Wuhan 430070, China; jkqiao@webmail.hzau.edu.cn (J.Q.); xuminghang@webmail.hzau.edu.cn (M.X.);; 2 The Cooperative Innovation Center for Sustainable Pig Production, Huazhong Agricultural University, Wuhan 430070, China

**Keywords:** immune cell, monocyte/lymphocyte ratio, neutrophil/lymphocyte ratio, pig, genome-wide association study, gene

## Abstract

Understanding the pig immune function is crucial for disease-resistant breeding and potentially for human health research due to shared immune system features. Immune cell ratios, like monocyte/lymphocyte ratio (MLR) and neutrophil/lymphocyte ratio (NLR), offer a more comprehensive view of immune status compared to individual cell counts. However, research on pig immune cell ratios remains limited. This study investigated MLR and NLR in a Duroc × Erhualian F_2_ resource population. Heritability analysis revealed high values (0.649 and 0.688 for MLR and NLR, respectively), suggesting a strong genetic component. Furthermore, we employed an ensemble-like GWAS (E-GWAS) strategy and functional annotation analysis to identify 11 MLR-associated and 6 NLR-associated candidate genes. These genes were significantly enriched in immune-related biological processes. These findings provide novel genetic markers and candidate genes associated with porcine immunity, thereby providing valuable insights for addressing biosecurity and animal welfare concerns in the pig industry.

## 1. Introduction

The immune system, a complex network of immune organs, immune cells, and immune molecules, plays a crucial role in executing immune responses and maintaining the stability of the body’s internal environment and physiological equilibrium [1,2]. Within this system, immune cells, commonly referred to as leukocytes, serve as key players in various immunological processes [3]. These cells, including lymphocyte, monocyte, granulocyte, and other cellular components, form the cellular foundation of the immune response [4]. Immune cell parameters can serve as an immunological index for livestock, reflecting their health status [5].

The pig industry faces urgent challenges related to biosafety and animal welfare caused by various infectious diseases [6,7]. Investigating immune-cell traits in pigs contributes significantly to genetic improvements in health and immune traits, thereby promoting biological safety prevention and control and enhancing disease-resistant breeding [8]. Additionally, due to their remarkable anatomical and immunological similarities to humans, pigs are widely used as animal models in human disease research [9]. Studying immune-cell traits in pigs can therefore contribute to our understanding of human diseases.

The immune response relies on the coordinated action of various immune cells. Immune cells ratios, emerging biomarkers in recent years, can combine the immune response of various immune cells. Recent research suggests that ratios of these immune cells, rather than individual immune cell counts, offer a more comprehensive view of the immune system’s health [10,11,12,13,14]. Among these emerging biomarkers, the monocyte/lymphocyte ratio (MLR) and neutrophil/lymphocyte ratio (NLR) can capture the synergistic interplay between innate and adaptive immunity [15,16,17]. Monocytes and neutrophils are integral components of the second line of defense against pathogen invasion, playing a pivotal role in the innate immune response. Neutrophils possess the ability to phagocytize pathogens, and their chemotaxis can reflect the state of the innate immune system [18]. Monocytes possess robust phagocytic capabilities and are capable of stimulating lymphocytes and other immune cells [19]. Additionally, the involvement of lymphocytes in the immune system’s third line of defense is characterized by their ability to specifically recognize pathogens and actively participate in both cellular and humoral immune responses [20]. Therefore, the MLR and NLR can offer a more precise assessment of the overall immune system status compared to individual cell counts. Analyzing the MLR and NLR provides a more nuanced understanding of the immune system by capturing the balance between different cell types, potentially revealing subtle changes that might be missed by examining individual cell counts alone.

Genome-wide association study (GWAS) has been extensively employed in the analysis of complex traits to identify associations between molecular markers and traits [21,22]. Past GWAS success in analyzing immune traits demonstrates its potential for elucidating the genetic architecture underlying immune traits [23,24,25,26,27]. A series of GWAS models were developed based on various genetic or statistical hypotheses [28,29,30,31]. However, given the diversity in the genetic architecture of complex traits, no single GWAS model is universally applicable. Combining multiple models has become an increasingly utilized approach to analyzing complex traits [32,33,34,35].

Previous studies have identified numerous quantitative trait loci (QTL) associated with immune-cell traits in pigs (https://www.animalgenome.org/cgi-bin/QTLdb, accessed on 20 May 2024). For example, 111 QTL on chromosomes 4, 6, 12, 13, and 17 have been linked to the neutrophil count. Similarly, approximately 39 QTL have been associated with the monocyte count, and around 112 QTL with the lymphocyte count. However, research on the genetic associations of the MLR and NLR in pigs remains limited. This limitation greatly hinders our understanding of the genetic mechanism underlying immune cells in pigs. 

To comprehensively understand the porcine immune response, this study conducted a GWAS analysis on two immune cell-derived traits (MLR and NLR) in a Duroc × Erhualian F_2_ resource population. Finally, we identified potential single-nucleotide polymorphisms (SNPs) and candidate genes associated with these immune cell ratios. This research will facilitate the genetic improvement of immune traits in pigs and offer useful information for disease-resistant breeding within the pig industry. 

## 2. Results

### 2.1. MLR and NLR of Duroc × Erhualian F2 Resource Population Exhibited High Heritability

The genetic variance, residual variance, and heritability of the NLR and MLR were estimated. All estimated genetic parameters are presented in Table 1. Our analysis revealed high heritability for both MLR and NLR traits in the Duroc × Erhualian F_2_ resource population. The heritability estimates were 0.649 for the MLR and 0.688 for the NLR. 

### 2.2. GWAS Analysis Identified Five SNPs Associated with MLR Trait

**Figure 1:**
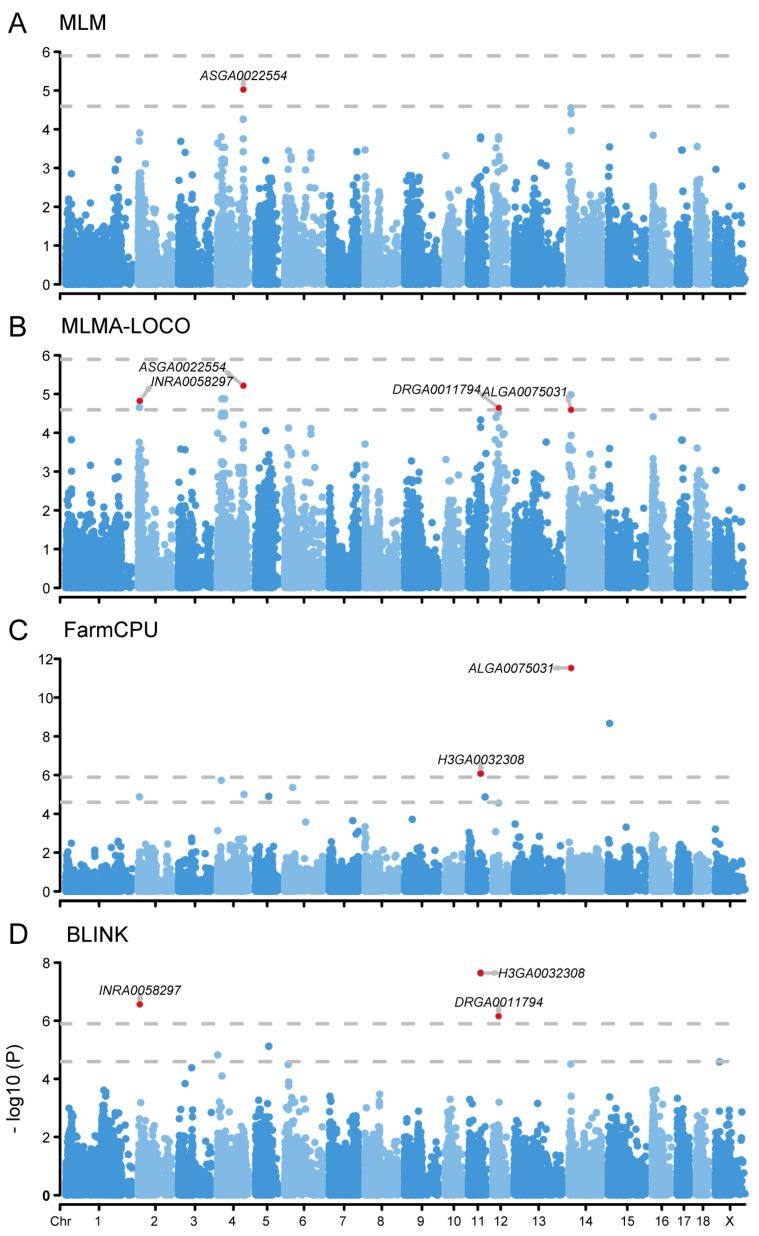
Manhattan plots of different GWAS models for MLR. (**A**), Manhattan plot of MLM model. (**B**), Manhattan plot of MLMA-LOCO model. (**C**), Manhattan plot of FarmCPU model. (**D**), Manhattan plot of BLINK model. The rows represent the GWAS results of different models, and the annotated SNPs are the ones identified by the E-GWAS. The dotted lines represent the Bonferroni correction thresholds.

The E-GWAS analysis identified five genome-wide SNPs associated with the MLR trait (Figure 1, Table 2). The genetic marker H3GA0032308, located on chromosome 11, exceeded the significance threshold in both FarmCPU and BLINK models. Based on the gene annotation information from the *Sus scrofa* genome, we identified the SLIT and NTRK like family member 1 (*SLITRK1*) gene located close to the SNP (Table S1). The SNPs INRA0058297 on chromosome 2 and DRGA001794 on chromosome 12 surpassed the significance threshold in the BLINK model and exceeded the suggestive threshold in the MLMA-LOCO model. Notably, the DRGA001794 falls within the range of the QTL that has been reported to exhibit significant association with the trait of the monocyte count (https://www.animalgenome.org/cgi-bin/QTLdb). Further, we identified 26 and 8 genes based on the two SNPs, respectively (Table S1). INRA0058297 on chromosome 14 was determined using FarmCPU and MLMA-LOCO models, and two genes, cell division cycle associated 2 (*CDCA2*) and EBF transcription factor 2 (*EBF2*), were identified in the *Sus scrofa* genome (Table S1). *CDCA2* is located approximately 5kb upstream of the SNP, while *EBF2* is located about 246kb downstream of the SNP. We also identified ASGA0022554 on chromosome 4 using the MLM and MLMA-LOCO models, but no genes were found within a 500kb range upstream and downstream of this SNP.

### 2.3. GWAS Analysis Identified Four SNPs Associated with NLR Trait

**Figure 2:**
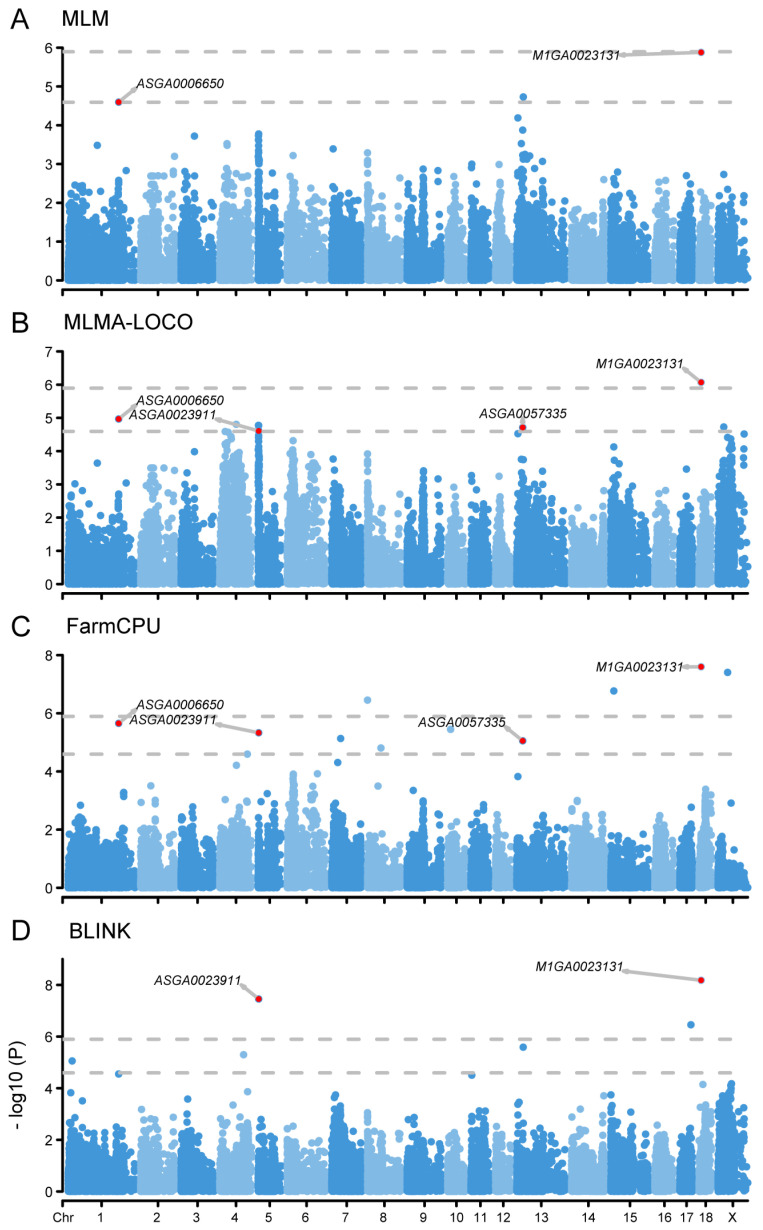
Manhattan plots of different GWAS models for NLR. (**A**), Manhattan plot of MLM model. (**B**), Manhattan plot of MLMA-LOCO model. (**C**), Manhattan plot of FarmCPU model. (**D**), Manhattan plot of BLINK model. The rows represent the GWAS results of different models, and the annotated SNPs are the ones identified by the E-GWAS. The dotted lines represent the Bonferroni correction thresholds.

The E-GWAS analysis for the NLR trait identified four associated SNPs located on chromosomes 1, 5, 13, and 18 (Figure 2, Table 3). The SNP M1GA0023131, located on chromosome 18, exhibited a significant association with the trait across all four models. The genetic variant ASGA0023911 on chromosome 5 surpassed the threshold in the BLINK model and exceeded the suggestive threshold in both MLMA-LOCO and FarmCPU models. Based on the suggestive threshold, two SNPs, ASGA0006650 and ASGA0057335, were also detected to be associated with the NLR trait. Among these, ASGA0006650 was detected in the MLM, MLMA-LOCO, and FarmCPU models, and ASGA0057335 was detected in MLMA-LOCO and FarmCPU. Within a 500kb range upstream and downstream of these four SNPs, we further identified 3, 5, 3, and 8 genes, respectively (Table S2).

### 2.4. Functional Enrichment Analysis Identified Multiple Candidate Genes for MLR and NLR

**Figure 3:**
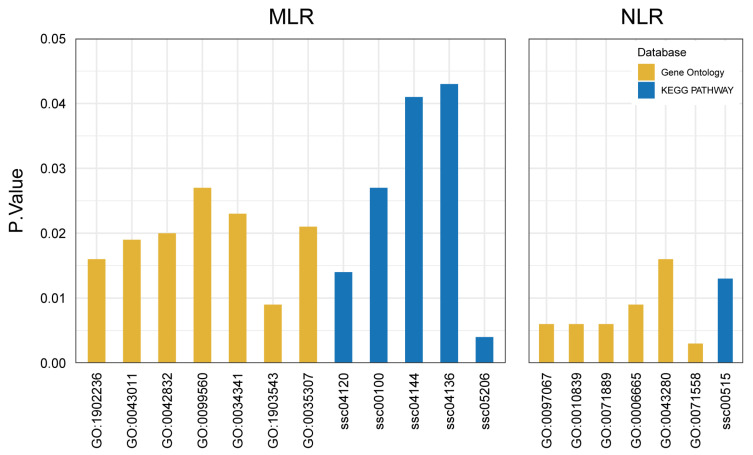
Immune-related biological processes in enrichment analysis results. The *X*-axis represents the ID of the enriched term, and the *Y*-axis represents the significant *p* value.

GO and KEGG enrichment analyses were performed on 37 MLR-related genes and 19 NLR-related genes (Table S3). The results revealed the involvement of multiple genes in immune-related biological processes, including the defense response to protozoan, the response to interferon-gamma, MicroRNAs in cancer, the sphingolipid metabolic process, autophagy and endocytosis (Figure 3, Table S3). We further consulted relevant PubMed literature (https://pubmed.ncbi.nlm.nih.gov/, accessed on 25 May 2024) and identified 11 and 6 candidate genes related to MLR and NLR traits, respectively (Table 4).

## 3. Discussion

The maintenance of porcine health relies on the coordinated action of multiple immune cells. While numerous GWAS analyses have been conducted to identify QTL associated with individual immune-cell traits in pigs [36,37,38,39], our study focuses on the ratios between different immune cell types. These ratios, specifically the MLR and NLR, provide a more comprehensive view of the immune response by combining the actions of distinct immune cells [10,11,12]. Applying E-GWAS to the Duroc × Erhualian F_2_ resource population, we successfully identified 5 SNPs associated with the MLR and 4 SNPs associated with the NLR. 

Most immune-cell traits are heritable. Research has shown that most immune-cell traits in pigs exhibit moderate to high heritability, with estimates ranging from 0.4 to 0.8 [40,41,42]. Our study corroborates and extends these findings, revealing high heritability for both MLR and NLR traits in the Duroc × Erhualian F_2_ resource population. These results provide robust evidence for the significant genetic influence on the MLR and NLR, reinforcing the potential for genetic improvement of these traits through selective breeding. 

The present study successfully identified a total of 11 candidate genes associated with the MLR. Notably, *IGF2BP1* (insulin-like growth factor 2 mRNA binding protein 1), *MIR192* (microRNA mir-192), and *CACD5* (cell division cycle associated 5) are significantly enriched in the KEGG pathway of MicroRNAs in cancer. Among them, *IGF2BP1* has been reported to modulate both innate and adaptive immune responses [43]. Similarly, the *MIR192* gene, a crucial component of exosomes, regulates gene expression and mediates the host’s antiviral immune response [44]. A study has demonstrated that exosomes from simulated infected newborn piglets can inhibit porcine epidemic diarrhea virus infection [45]. Additionally, silencing the *CDCA5* gene has been shown to inhibit the AKT signaling pathway, activating the pro-apoptotic signaling pathway and revealing *CDCA5*’s functional role in hepatocellular carcinoma [46]. *SYVN1* (synoviolin 1) is significantly enriched in the ubiquitin-mediated proteolysis pathway, and defects in this system are linked to various diseases [47]. An analysis of miRNA expression profiles in lawsonia intracellularis-infected porcine intestines revealed an upregulation of *SYVN1* [48]. *TM7SF2* (transmembrane 7 superfamily member 2) on chromosome 4 is involved in steroid biosynthesis, with steroids known to have immunosuppressive effects [49,50]. *SLITRK1* (SLIT and NTRK like family member 1) function changes may be implicated in neuropsychiatric disorders [51]. *ATG2A* (autophagy-related 2A) is significantly enriched in the biological process of autophagy, an essential pathway for immune balance [52]. The *CALCOCO2* (calcium binding and coiled-coil domain 2) gene responds to interferon-gamma and regulates pro-apoptotic and autophagy-related genes [53]. The *SNF8* (SNF8 subunit of ESCRT-II) participates in interferon-mediated antiviral responses [54,55]. *CDCA2* (cell division cycle associated 2), a member of cell cycle-related proteins, regulates cell proliferation and is involved in the development of various cancers [56,57,58]. Lastly, *BATF2* (basic leucine zipper ATF-like transcription factor 2) plays a crucial role in the innate immune response and defense against protozoan infections [59].

Our NLR analysis identified six potential candidate genes, each with distinct roles in immune-related processes. Among these genes, the *POMGNT2* (protein O-linked mannose N-acetylglucosaminyltransferase 2 (beta 1, 4-)) gene is involved in the biosynthesis of Mannose type O-glycans which can participate in hematopoiesis and inflammatory responses [60,61]. It was identified as a candidate gene for a significantly reduced total number born by a separate study on lethal recessive mutations in pigs [62]. The *KDM7A* (lysine demethylase 7A) gene functions in post-translational modification and has been linked to cancer development and inflammatory responses [63,64]. The *TBC1D22A* (TBC1 domain family member 22A) gene interacts with 14-3-3 proteins, which play a crucial role in various neurological diseases [51]. The *KLF9* (KLF transcription factor 9) gene is significantly enriched in the biological processes of the cellular response to thyroid hormone stimulus and negative regulation of keratinocyte proliferation. The thyroid hormone is believed to maintain immune cell activity and function [65,66,67]. Additionally, keratinocytes contribute significantly to innate immune responses [68]. The *GRAMD4* (GRAM domain containing 4) gene positively regulates cysteine-type endopeptidase activity in the apoptotic process. Lastly, *CERK* (ceramide kinase) is involved in the sphingolipid metabolic process, and the regulatory mechanisms of key sphingolipids affect various biological processes such as inflammation, cellular proliferation, and apoptosis [69,70]. 

While our findings provide valuable insights for the investigation of immune responses in pigs as well as genetic diseases relevant to humans, they also highlight the need for further study. Most of the identified genes, though known to be involved in immune regulation, require additional functional characterization to elucidate their precise mechanisms of action in both porcine and human contexts. Future studies focusing on the functional validation of these genes will provide a comprehensive and robust explanation for this statistical analysis, further enhancing our understanding of immune mechanisms in both pigs and humans.

## 4. Materials and Methods

### 4.1. Animals 

The study used an F_2_ resource population (393 pigs) resulting from crossbreeding Duroc and Erhualian [8]. A total of 8 Duroc boars were crossed with 18 Erhualien sows, and subsequently, 31 boars and 38 sows from the F_1_ generation were selected for mating to obtain the F_2_ population. All animals were raised at the experimental facility operated by a prominent breeding company. The ear or tail tissues of the F_2_ hybrids were collected.

### 4.2. Phenotype

It has been demonstrated that 7-week-old pigs possess a fully developed immune system, rendering them suitable as an animal model for immunological investigations [71]. In this study, blood samples (1 mL) were collected from the jugular vein of the piglets using vacuum tubes containing EDTA-K2 at the age of 35 days. Subsequently, blood parameters were measured using a photoelectric MEK-8222K fully automatic five-category blood cell analyzer (Nihon Kohden, Tokyo, Japan) at the People’s Hospital of Xinxing County, Yunfu, Guangdong, China [8]. The MLR and NLR were computed based on three blood parameters, namely the monocyte count, lymphocyte count, and neutrophil count, for subsequent analysis.

### 4.3. Genotype

The DNA samples were extracted from ear or tail tissues (336 pigs), followed by genotyping using an iScan system (Illumina Inc., San Diego, CA, USA) with PorcineSNP60 BeadChips, resulting in a total of 62,163 SNPs. SNPs with a missing rate higher than 0.05 underwent quality control using Plink (v1.9) [72], while the remaining genotypes were imputed using Beagle (v5.4) [73]. After imputation, SNPs with a minor allele frequency (MAF) < 0.01 were excluded [72]. Following genotype data processing, a total of 39,292 SNPs were used for analysis.

### 4.4. Estimation of Genetic Variance and Heritability

The genetic variance and heritability were estimated through restricted maximum likelihood (REML) analysis with GCTA software (v1.94.1) [29]. The model of estimating variance can be written as:

y=Xb+Zu+e

where y is the vector of trait (including MLR and NLR); b is the fixed effects; u denotes the additive genetic effect, with a normal distribution $u\sim N(0,G\sigma_{u}^{2})$; X and Z are respective incidence matrices for the fixed effect and additive genetic effect, respectively; *e* is the residual error following a normal distribution $e\sim N(0,I\sigma_{e}^{2})$, where *I* indicates the identity matrix. Heritability ($h^{2}$) was estimated using the formula: $h^{2}=\sigma_{u}^{2}/\sigma_{y}^{2}$, where $\sigma_{u}^{2}$ and $\sigma_{y}^{2}$ represent the additive genetic variance and phenotypic variance, respectively.

### 4.5. Genome-Wide Association Study

E-GWAS can efficiently integrate diverse GWAS models to yield more robust and dependable outcomes [74]. In the analysis, the mixed-effect linear model (MLM) [28], MLM leaving-one-chromosome-out (MLMA-LOCO) [29], fixed and random model circulating probability unification (FarmCPU) [30], and Bayesian-information and linkage-disequilibrium iteratively nested keyway (BLINK) [31] models were integrated by the E-GWAS strategy to conduct genome-wide association studies (GWASs). 

The MLM model controls false positives by stratifying groups as a fixed effect and using the individual relationship matrix as a random effect [28]. The MLM model was implemented using GCTA software, with the following formula:

y=Xb+Sd+Zu+e

where *y* is the phenotype, *b* is the fixed effects; *d* is the additive effect of the candidate SNP to be tested for association; *u* is the additive genetic effect, with a normal distribution $u\sim N(0,G\sigma_{u}^{2})$, the *G* was calculated using all SNPs; *X* and Z are respective incidence matrices for *b* and *u*; *S* is the genotype indicator variable of the candidate SNP to be tested, coded as 0, 1, or 2; *Z* is the incidence matrices for *u*; *e* is the residual error following a normal distribution $e\sim N(0,I\sigma_{e}^{2})$, where *I* indicates the identity matrix.

The MLMA-LOCO model builds upon the MLM by excluding the influence of SNPs on the chromosome where the candidate SNP is located, further improving accuracy [29]. The MLMA-LOCO model was performed using GCTA software, with the following formula:

$$y=Xb+Sd+Zu^{-}+e$$

where *y*, *b*, *d*, *e*, *X*, *S*, and *Z* are the same as those in the MLM model; $u^{-}$ is the additive genetic effect, with the assumption that $u^{-}\sim N(0,{G^{-}\sigma }_{u^{-}}^{2})$; the $G^{-}$ was calculated using all SNPs except those on the chromosome where the candidate SNP is located; the $\sigma_{u^{-}}^{2}$ will be re-estimated each time when a chromosome is excluded from the $G^{-}$ calculation.

The FarmCPU model improves SNP detection by dividing the MLM into a separate fixed-effects model (FEM) and random-effects model (REM), and iteratively utilizing both models [30]. The FarmCPU model was implemented using the rMVP (v1.0.6) package [75]. 

The FEM was calculated based on the following formula:

y=Xb+Mp+Sd+e

where *y*, *b*, *d*, *e*, *X*, and *S* are the same as those in the MLM model; *M* is the genotype matrix of pseudo-SNPs that are used as fixed effects; *p* is the relevant design matrix for *M*. 

The REM was employed to select the most suitable pseudo-SNPs, as follows:

y=g+e

where *y* and *e* are the same as those in the FEM; *g* is the additive genetic effect, with a normal distribution $g\sim N(0,K\sigma_{g}^{2})$, the *K* was calculated using pseudo-SNPs.

The BLINK model replaces the REM in FarmCPU with the FEM based on Bayesian information criteria, and substitutes the bin method of FarmCPU with linkage disequilibrium information, significantly reducing the computational time while improving the statistical power [31]. The BLINK model was built using the BLINK (v1.0.6) package, with the following formula:

The first FEM: y=Mp+Sd+e

The second FEM: y=Mp+e

where *y*, *p*, *d*, *e*, *M*, and *S* are the same as those in the FarmCPU model. The two FEM models have two differences: firstly, the second FEM excludes the testing marker in the first FEM; secondly, the number of covariate pseudo QTNs varies in the second FEM to select an optimal set of k out of t pseudo QTNs.

A Bonferroni correction was applied to establish the threshold for significance [76]. To avoid overlooking potential linkage signals, the genome-wide levels of significance and suggestiveness thresholds were defined as *p* = 0.05/*N* and *p* = 1/*N*, respectively, where *N* represents the number of SNPs.

### 4.6. Identification and Functional Analysis of Candidate Genes

The biomaRt (v2.58.2) package [77,78] was employed to identify genes associated with the MLR and NLR within a 500 kb region upstream and downstream of identified SNPs by GWAS. Subsequently, we performed Kyoto Encyclopedia of Genes and Genomes (KEGG) analysis and gene ontology (GO) enrichment analysis using the KOBAS 3.0 website (http://kobas.cbi.pku.edu.cn, accessed on 25 May 2024) [79,80].

## 5. Conclusions

Our analysis of the MLR and NLR in the Duroc × Erhualian F_2_ resource population has successfully identified several SNPs and candidate genes associated with these immune cell ratios. These findings offer valuable insights into the underlying genetic mechanisms of immune regulation in pigs. This knowledge can be leveraged to develop breeding programs for disease resistance, a significant goal within the pig industry. Furthermore, the identified candidate genes have been implicated in human disease development and immune processes. Since pigs are a well-established animal model for human diseases, our findings also offer valuable reference for human disease research.

## Figures and Tables

**Table 1:** Estimation of variance components and heritability for mononuclear-lymphocyte ratio (MLR), and neutrophil-lymphocyte ratio (NLR).

Trait	^1^ $\sigma_{u}^{2}$ ± SE	^2^ $\sigma_{e}^{2}$ ± SE	^3^ $h^{2}$ ± ^4^ SE
MLR	0.008 ± 0.002	0.004 ± 0.001	0.649 ± 0.080
NLR	1.384 ± 0.273	0.627 ± 0.118	0.688 ± 0.076

^1^ $\sigma_{u}^{2}$, additive genetic variance; ^2^ $\sigma_{e}^{2}$, dominance genetic variance; ^3^ $h^{2}$, additive heritability; ^4^ SE, standard error.

**Table 2:** SNPs associated with monocyte/lymphocyte ratio (MLR).

SNP	^1^ Chr	Position	Model	^2^ *p*-Value
INRA0058297	2	7110358	MLMA-LOCO	1.51 × 10^−5^
			BLINK	2.71 × 10^−7^ *
ASGA0022554	4	116094650	MLM	9.41 × 10^−6^
			MLMA-LOCO	6.07 × 10^−6^
H3GA0032308	11	54602515	FarmCPU	8.25 × 10^−7^ *
			BLINK	2.27 × 10^−8^ *
DRGA0011794	12	25068363	MLMA-LOCO	2.28 × 10^−5^
			BLINK	6.92 × 10^−7^ *
ALGA0075031	14	9571032	MLMA-LOCO	2.54 × 10^−5^
			FarmCPU	2.98 × 10^−12^ *

^1^ Chr, Chromosome; ^2^ *p*-value, *p* values of different GWAS models are represented by * above the significant threshold and without * above the suggestiveness threshold.

**Table 3:** SNPs associated with neutrophil/lymphocyte ratio (NLR).

SNP	^1^ Chr	Position	Model	^2^ *p*-Value
ASGA0006650	1	223397796	MLM	2.53 × 10^−5^
			MLMA-LOCO	1.08 × 10^−5^
			FarmCPU	2.21 × 10^−6^
ASGA0023911	5	2488153	MLMA-LOCO	2.47 × 10^−5^
			FarmCPU	4.67 × 10^−6^
			BLINK	3.50 × 10^−8^ *
ASGA0057335	13	26494790	MLMA-LOCO	1.94 × 10^−5^
			FarmCPU	8.84 × 10^−6^
M1GA0023131	18	9230153	MLM	1.32 × 10^−6^ *
			MLMA-LOCO	8.52 × 10^−7^ *
			FarmCPU	2.52 × 10^−8^ *
			BLINK	6.60 × 10^−9^ *

^1^ Chr, Chromosome; ^2^ *p*-value, *p* values of different GWAS models are represented by * above the significant threshold and without * above the suggestiveness threshold.

**Table 4:** Summary of candidate genes associated with monocyte/lymphocyte ratio (MLR) and neutrophil/lymphocyte ratio (NLR).

Trait	Gene	^1^ Chr	^2^ Term	Database	ID	^3^ *p*-Value
MLR	*SYVN1*	2	Ubiquitin mediated proteolysis	KEGG PATHWAY	ssc04120	0.014
			negative regulation of endoplasmic reticulum stress-induced intrinsic apoptotic signaling pathway	Gene Ontology	GO:1902236	0.016
	*TM7SF2*	2	Steroid biosynthesis	KEGG PATHWAY	ssc00100	0.027
	*CDCA5*	2	MicroRNAs in cancer	KEGG PATHWAY	ssc05206	0.004
	*BATF2*	2	Endocytosis	KEGG PATHWAY	ssc04144	0.041
			myeloid dendritic cell differentiation	Gene Ontology	GO:0043011	0.019
			defense response to protozoan	Gene Ontology	GO:0042832	0.020
	*ATG2A*	2	Autophagy—other	KEGG PATHWAY	ssc04136	0.043
	*MIR192*	2	MicroRNAs in cancer	KEGG PATHWAY	ssc05206	0.004
	*SLITRK1*	11	synaptic membrane adhesion	Gene Ontology	GO:0099560	0.027
	*CALCOCO2*	12	response to interferon-gamma	Gene Ontology	GO:0034341	0.023
	*SNF8*	12	positive regulation of exosomal secretion	Gene Ontology	GO:1903543	0.009
	*IGF2BP1*	12	MicroRNAs in cancer	KEGG PATHWAY	ssc05206	0.004
	*CDCA2*	14	positive regulation of protein dephosphorylation	Gene Ontology	GO:0035307	0.021
NLR	*KLF9*	1	cellular response to thyroid hormone stimulus	Gene Ontology	GO:0097067	0.006
			negative regulation of keratinocyte proliferation	Gene Ontology	GO:0010839	0.006
	*TBC1D22A*	5	14-3-3 protein binding	Gene Ontology	GO:0071889	0.006
	*CERK*	5	sphingolipid metabolic process	Gene Ontology	GO:0006665	0.009
	*GRAMD4*	5	positive regulation of cysteine-type endopeptidase activity involved in apoptotic process	Gene Ontology	GO:0043280	0.016
	*POMGNT2*	13	Mannose type O-glycan biosynthesis	KEGG PATHWAY	ssc00515	0.013
	*KDM7A*	18	histone demethylase activity (H3-K27 specific)	Gene Ontology	GO:0071558	0.003

^1^ Chr, Chromosome; ^2^ Term, biological processes of enrichment analysis, ^3^ *p*-Value, *p* value of enrichment analysis.

## Data Availability

The data analyzed in this study are accessible on figshare (https://figshare.com/s/daf50a666ff6c3d8514f, accessed on 5 June 2024).

## Supplementary Materials

The following supporting information can be downloaded at: https://www.mdpi.com/article/10.3390/ijms25179745/s1.
